# Variability in daily or weekly working hours and self-reported mental health problems in Korea, Korean working condition survey, 2017

**DOI:** 10.1186/s13690-021-00545-z

**Published:** 2021-02-27

**Authors:** Hye-Eun Lee, Myoung-Hee Kim, Min Choi, Hyoung-Ryoul Kim, Ichiro Kawachi

**Affiliations:** 1 Korea Institute of Labor Safety and Health, Seoul, Republic of Korea; 2 Department of Social and Behavioral Sciences, Harvard T.H. Chan School of Public Health, Boston, MA USA; 3 People’s Health Institute, Seoul, Republic of Korea; 4 Department of Occupational and Environmental Medicine, College of Medicine, The Catholic University of Korea, Seoul, Republic of Korea

**Keywords:** Self-reported anxiety symptoms, Self-reported depressive symptoms, Flexibility, Variability, Work schedule

## Abstract

**Background:**

Working hour regulation in Korea is being revised to allow increasing variability in number of working hours. We sought to investigate the association between variability in the number of daily or weekly working hours with or without long working hours (> 52 h/w) and mental health among South Korean workers.

**Methods:**

We used data from 28,345 full-time, non-shift employed workers working more than 30 h per week participating in the Korean Working Condition Survey in 2017. We defined six groups according to variability in daily or weekly working hours (same number vs different number) and weekly working hours (31–40, 41–52, > 52 h per week). Odds ratios (ORs) and confidence intervals (CIs) for self-reported depressive symptoms and anxiety were calculated using workers with same number of working hours/31–40 h per week as the reference.

**Results:**

Variability in number of work hours every day or week combined with > 52 working hours per week showed the highest risk of depressive symptoms (OR = 5.13, 95% CI 3.25–8.11) and anxiety (OR = 3.75, 95% CI 2.39–5.88) compared to the reference group, controlling for age, sex, education, occupation, industry, salary, workers’ choice of working hours and overtime payment. Workers working ≤52 h/w were adversely impacted by variable working hours as well.

**Conclusions:**

Variable daily or weekly working hours were associated with poorer self-reported depressive and anxiety symptoms in Korea, among full-time and non-shift workers. Reform of the Korean Labor Standards Act warrants consideration.

**Supplementary Information:**

The online version contains supplementary material available at 10.1186/s13690-021-00545-z.

## Background

Long working hours is a social determinant of workers’ wellbeing [1]. As a country with the some of the longest recorded working hours, South Korea has sought to restrict the maximum working hours through legislation. The Labor Standards Act in Korea (covering employed workers in the workplace with 5 or more workers) determined that 40 h per week is the standard work hours and 52 h is the permissible maximum with the worker’s consent. The legislation does not apply to several types of occupations due to exceptions (e.g. surveillance workers, agriculture and fishery workers, transportation workers, or health care workers) nor to employees of small sized workplace (fewer than five employees); nonetheless, the working hour regulations represents a basic protective measure for long working Korean workers. However, working on weekends was not subject to regulation until 2018, and hence many workers were doing up to 68 h per week. In 2018, the Korean government amended the law to include weekends. The ‘Korea Enterprise Federation’ representing employers responded to this decision by demanding an expansion of the ‘flexible work hour system’, specifically lengthening the reference period for calculating the average weekly working hours for up to 1 year. After intense negotiations, a council consisting of representatives from labor, employers, and the government agreed to revise the reference period up to 6 months (from the previous 3 months). According to this agreement, workers may work up to 64 h per week for 3 months as long as they work 40 h per week for the remaining 3 months. Therefore, workers have become concerned about overwork from compressed work schedule as well as irregular, unpredictable work schedules, even though the maximum permissible working hours decreased on average. With expansion of ‘flexible work hour system’, the start and end time of work is more likely to fluctuate (i.e., irregular work schedules) and this variability is more likely to be driven by the needs of employer, and thus difficult to predict for the workers.

Flexibility in working hours was already a rising phenomenon since the 1990s in developed countries attempting to adjust to the “24 hour Society” [2–4]. Several studies have explored the impact of flexible working hours on workers’ health in the Western setting, where average working hours have been much shorter than in Korea. According to previous studies, there is an obvious distinction between company-oriented flexibility and worker-oriented flexibility [2, 5]. Generally speaking, flexible working hours initiated by employers (and motivated by organizational interests) have shown a negative impact on workers’ mental health [2, 6] and work-life conflict [7–9], whereas worker-initiated flexible working hours have been found to have either a positive or equivocal impact on health in the Western setting [3, 5].

However, there have been few studies investigating the association between flexibility in working hours and workers’ health in Korea. A previous study on Korean workers’ working conditions and depressive symptoms reported that precarious jobs with unfixed work hours were associated with a higher risk of the depressive symptoms compared to day time workers [10]. Both precarious work and shift work have been independently shown to affect workers’ health, and may therefore confound the association between unfixed work hours and health. Hence there is a need to study full-time, non-shift workers.

Some employers and politicians have argued that flexibility in work hours is beneficial for workers by enhancing work-life balance [5]. Regulatory changes for flexible work hours and long working hours continue to be in a state of flux, yet empirical evidence on their health effects remains sparse.

Therefore, the purpose of the current study was to investigate the relation between variability in daily or weekly working hours with or without long working hours (> 52 h/w) and mental health based on a sample of full-time, non-shift workers in nation-wide survey data in Korea.

## Methods

### Study population

Data from the 5th Korean Working Conditions Survey (KWCS) in 2017 were used for analysis. The KWCS is a cross-sectional national survey regularly administered by the Korea Occupational Safety and Health Agency (KOSHA) [11]. The target population of KWCS is the entire working population in Korea. The survey uses a stratified cluster sampling design based on the National Census Registry. In this survey, information on working conditions as well as physical and psychosocial health are collected from an interview conducted by professional interviewers. A previous study reported that KWCS showed high external and content validity and reliability [11].

**Fig. 1:**
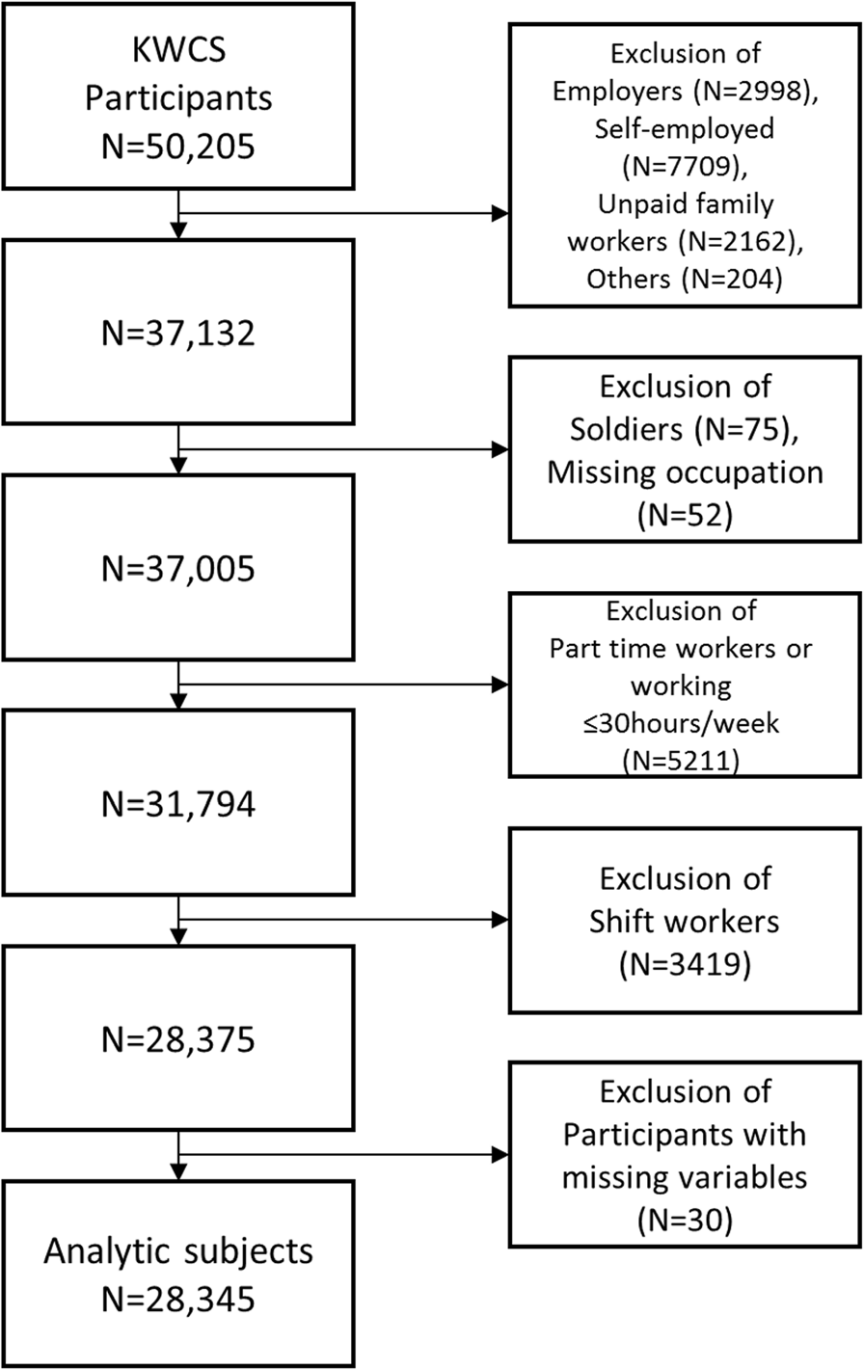
Flow chart illustrating the process of creating the analytic sample, Korean Working Condition Survey, 2017. KWCS = Korean Working Condition Survey

Among 50,205 participants in the 2017 KWCS wave, we restricted subjects to full-time employed workers working more than 30 h per week. We also excluded the following individuals: soldiers or missing occupation, shift workers, individuals with missing information on the variables used in the analysis. After these exclusions, our analytic sample comprised 28,345 participants. The analytic sample selection process is depicted in Fig. 1. Part-time workers and individuals who worked less than 30 h per week were excluded from the analysis to control the confounding effects of underemployment.

### Mental health

Mental health was assessed by two questions. If the participants chose the response option “yes” to the questions “Over the last 12 months, did you have depressive symptoms?” and “Over the last 12 months, did you have anxiety?”, they were defined to have depressive symptoms and anxiety, respectively. If they chose “no” to the questions, they were considered to have no symptoms, and the participants who chose “don’t know” were excluded from the analysis.

### Total working hours and variability in working hours

Working hours was assessed by a question on the KWCS, asking: “How many hours do you usually work per week in your main paid job?” Working hours were classified into three groups: (i) 31–40 h, (ii) 41–52 h, and (iii) > 52 h per week. This classification is based on the Labor Standard Act in Korea, in which 40 h is defined as the standard work week, and 52 h is the maximum permitted working hours per week [12].

Daily or weekly working hours were considered variable if the participants responded “no” to any of the following questions; (i) “Do you work the same number of hours every day?”, (ii) “Do you work the same number of days every week?”, and (iii) “Do you work the same number of hours every week?” We classified subjects into six groups according to combinations of variability in working hours and total weekly working hours.

### Covariates

Age, sex, education, occupation, industry, salary and workers’ choice of the working hour were included in our regression models as possible confounders. All this information was collected from the interviews during the survey.

Occupation was coded into 10 categories according to the Korean Standard Classification of Occupation [13], and industry was coded into 21 categories according to the Korean Standard Industrial Classification [14]. Workers’ choice of working hours was assessed by the question “How are your working hours determined?” Response options included: (i) “They are set by the company/organization with no possibility for change” (labeled was as ‘rigid’), (ii) “You can choose between several fixed working schedules determined by the company/organization”, (iii) “You can adapt your working hours within certain limits (e.g. flextime)”, or (iv) “Your working hours are entirely determined by yourself” (labeled as “free choice”). Overtime payment was defined as yes when the participants responded positively to the question “Do your earnings from your main job include extra payments for additional hours of work/overtime?”

### Statistical analysis

We used the sampling weights from the KWCS in the analysis to make the findings nationally representative. Depressive symptoms and anxiety were compared across the groups classified by working hours, irregular working hours and other sociodemographic and occupational factors. A multiple logistic regression analysis was conducted to examine the relationship between irregular working hours and mental health using the group with regular working hours reporting 31–40 h/w as the reference group. Multiple logistic regression models were adjusted for age, sex, education, occupation, industry, salary, workers’ choice of the working hour, and overtime payment. We formally tested for interaction between irregular working hours x gender, which was found to be statistically significant (*p* = 0.0094), hence all analyses were stratified by gender. The significance level for all statistical analyses was *p* < 0.05 using a two-tailed test. SAS version 9.4 (SAS Institute, Cary, NC, USA) was used for statistical analysis.

## Results

Characteristics of the study sample and the prevalence of depressive symptoms and anxiety are summarized in Table 1. There were 5066 workers (17.9%) who reported variability in daily or weekly working hours. More than half of the respondents (56.2%) worked 31-40 h/w, 8668 workers (30.6%) worked for 41-52 h/w, and 3743 workers (13.2%) worked for more than 52 h/w. The prevalence of depressive symptoms was 2.1% and that for anxiety was 2.7% in the total sample, and they were higher in those who had variable working hours and worked longer hours than in those who did not. The distribution of variable daily or weekly working hours according to sociodemographic factors and other occupational factors are presented in Supplementary Table S1.

**Table 1:** Characteristics of participants and prevalence of depressive symptom and anxiety, Korean Working Condition Survey, 2017

	Weighted frequency	%	Depressive symptom (%)	p	Anxiety (%)	p
Total	28,345	100.0	2.1		2.7	
Sex						
men	17,003	60.0	1.9	0.076	2.8	0.245
women	11,342	40.0	2.4		2.5	
Age						
< 30	4113	14.5	2.0	0.009	2.4	0.054
30–39	7601	26.8	1.5		2.1	
40–49	8055	28.4	2.6		2.9	
50–59	6068	21.4	2.2		3.3	
60-	2508	8.8	2.6		2.4	
Education						
< High school	1533	5.4	3.1	0.102	2.7	0.919
High school	7881	27.8	2.0		2.6	
College	18,931	66.8	2.1		2.7	
Occupation						
Manager	1076	3.8	2.5	0.395	2.2	0.932
Professional	3071	10.8	2.0		2.5	
Technicians and associate professionals	1889	6.7	2.4		2.2	
Clerk	9551	33.7	1.8		2.6	
Service	2986	10.5	2.2		3.0	
Sales worker	2614	9.2	2.2		2.9	
Agricultural, forestry or fishery	59	0.2	1.0		1.3	
Craft and related trades	2891	10.2	2.1		2.7	
Plant, machine operator or assembler	1980	7.0	2.2		3.0	
Elementary occupation	2229	7.9	3.0		2.7	
Working hour per week						
31–40	15,934	56.2	1.8	0.002	2.2	0.001
41–52	8668	30.6	2.4		3.2	
> 52	3743	13.2	2.9		3.4	
Variability in daily or weekly working hour						
No	23,279	82.1	1.6	<.0001	1.9	<.0001
Yes	5066	17.9	4.7		6.0	
Monthly salary						
< 200	6914	24.4	2.5	0.030	2.4	0.0001
(10,000 KRW)						
200–299	8847	31.2	2.0		2.3	
300–399	6892	24.3	1.5		2.3	
> 400	5692	20.1	2.5		3.9	
Workers’ choice of working hour						
Rigid	23,658	83.5	1.7	<.0001	2.1	<.0001
Free choice	4687	16.5	4.2		5.4	
Overtime pay						
No or N/A	15,170	53.5	1.7	<.0001	2.1	<.0001
Yes	13,175	46.5	2.6		3.3	

*The results were obtained by χ2 test

Associations between variability in hours worked by total weekly working hours and self-reported mental health problems stratified by gender are shown in Tables 2 and 3. In men, after controlling for socio-demographic and work-related factors, the odds ratio (OR) for depressive symptoms and anxiety were significantly higher in the variable daily or weekly working hours group than in the same number of working hours and 31–40 h/w group. The group engaged in variable daily or weekly working hours for > 52 h/w showed the highest risk of depressive symptoms in both men (OR = 7.00, 95% CI 3.64–13.43) and women (OR = 4.31, 95% CI 2.16–8.61). They also showed a higher risk of anxiety in both men (OR = 4.69, 95% CI 2.63–8.37) and women (OR = 3.21, 95% CI 1.49–6.94).

**Table 2:** Adjusted odds ratio (95% confidence interval) of depressive symptoms according to variability in daily or weekly working hours by total weekly working hours, Korean Working Condition Survey, 2017

Variability in daily or weekly working hours	Working hour per week		
	31–40	41–52	> 52
Total			
No	Reference	1.33 (0.96–1.83)	1.37 (0.88–2.13)
Yes	**2.57 (1.66–4.00)**	**2.75 (1.80–4.21)**	**5.13 (3.25–8.11)**
Men			
No	Reference	**2.31 (1.41–3.79)**	1.63 (0.79–3.34)
Yes	**4.16 (2.12–8.17)**	**5.17 (2.93–9.15)**	**7.00 (3.64–13.43)**
Women			
No	Reference	0.83 (0.54–1.26)	1.23 (0.71–2.13)
Yes	1.60 (0.97–2.64)	1.06 (0.44–2.59)	**4.31 (2.16–8.61)**

*Adjusted by age, sex (total sample), education, occupation, industry, salary and workers’ choice of working hour, overtime pay

**Table 3:** Adjusted odds ratio (95% confidence interval) of anxiety according to variability in daily or weekly working hours by total weekly working hours, Korean Working Condition Survey, 2017

Variability in daily or weekly working hours	Working hour per week		
	31–40	41–52	> 52
Total			
No	Reference	1.18 (0.88–1.57)	1.25 (0.83–1.87)
Yes	**2.34 (1.55–3.52)**	**3.07 (2.18–4.33)**	**3.75 (2.39–5.88)**
Men			
No	Reference	**1.69 (1.12–2.57)**	1.36 (0.76–2.46)
Yes	**3.27 (1.83–5.86)**	**5.18 (3.31–8.09)**	**4.69 (2.63–8.37)**
Women			
No	Reference	0.75 (0.49–1.14)	1.14 (0.66–1.98)
Yes	1.50 (0.91–2.47)	0.87 (0.49–1.57)	**3.21 (1.49–6.94)**

*Adjusted by age, sex (total sample), education, occupation, industry, salary and workers’ choice of working hour, overtime pay

## Discussion

We found that individuals who do not work the same number of hours every day or week have a higher risk of depressive symptoms and anxiety compared to those working same number of hours among non-shift, full-time Korean workers. Though variable daily or weekly working hours combined with long working hours showed the greatest risk for depressive symptoms and anxiety, even the men engaged in variable working hours within the legally permissible working hour limit (≤52 h/w) showed elevated risk.

Our finding is in line with previous reports on the impact of irregular working hours. A study using the 3rd European Survey on Working Conditions found that individuals who do not work for same number of hours per day and week and have no fixed starting and finishing times (i.e., irregular working hours) showed a significantly higher prevalence of stress (34.8%) and anxiety (9.5%) compared to workers with fixed regular working hours (22.9 and 5.4%, respectively) [2]. A study using the same data reported that workers with fixed starting and ending times of work reported less stress (OR = 0.56, 95% CI 0.51–0.61) and anxiety (OR = 0.54, 95% CI 0.46–0.63) compared to individuals with irregular working hours [6].

Precarious work arrangements such as involuntary part-time work is a major contributor to irregular working hours [4, 15]. Low socioeconomic status and job insecurity among precarious workers could account for the adverse impact of irregular work hours on workers’ health. However, we restricted the study sample to full-time workers working more than 30 h/w to minimize the confounding effect of precarious work.

The adverse impact of variable number of hours worked on mental health could be mediated by sleep disturbance. Previous European survey found that workers with variable working hours showed higher risk for sleep problems even without shift work [2]. Having more fixed starting and ending work time makes possible to keep a more stable daily basic life such as sleep and meal, and regular sleep time is known to be associated with quality of sleep [6, 16]. Irregular working hours are also associated with more work-life conflict [7, 8]. Because, irregular working hours can lead to a shift away from regular daily working rhythms to a continually changing pattern of working hours and free time, the resulting disruptions in social interactions and social isolation may contribute to a deterioration in mental wellbeing [17]. In addition, irregular work hours may lead to job dissatisfaction, which could be in the path between irregular working hours and psychological symptoms [7].

In terms of worker-oriented flexibility in working hours, most previous researches reported flexible working hours reduced work-life conflict, thus increased worker’s health and well-being [5, 6, 18–20]. A study using representative data of working population in Finland showed that company-controlled flexibility in working hours such as overtime work or weekend work had a negative association with the social and mental well-being of employees, however, individual-controlled flexibility such as flexitime or working hour banks canceled the negative effect [21]. Although a worker-oriented flexible working hour system is designed to be favorable to workers by increasing worker’s control and choice, several studies showed only a partial positive effect or no effect on health or well-being [22–25]. This might be because flexibility is generally restricted within limits set by the company and even mostly self-controlled working hours may intervene with social rhythms if it is too long or arranged largely in unsocial hours such as evenings or weekends [5]. In the current study, the workers having a choice of their working hours showed more than two times higher prevalence of depressive symptoms and anxiety compared to the others. This finding could be caused by the different working conditions in Korea compared to Western countries. In our data, workers having a choice of working hours worked longer hours (mean: 45.9 h/w) than the others (44.7 h/w) and variable daily or weekly working hours were more prevalent among them (36.2% vs 14.2%)(data not shown). It was hard to evaluate the positive impact of worker’s control of the working hours in the current study because it was closely associated with long working hours and variable daily or weekly working hours in Korea.

The most distinctive feature of Korean workers is their long working hours. Caution is warranted in drawing cross-national comparisons of the impact of working hour arrangements on health. For example, a study using representative data of a German working population reported that workers with ‘flexible extended work schedule’ (i.e., non-shift workers having high probability of overtime or weekend work and high working time control) showed better self-rated health compared to workers with ‘rigid standard work schedule’, though individuals with ‘flexible standard work schedule’ showed the highest self-rated health [26]. However, there is a substantial gap between the average working hours in Korea (1993 h/year) compared to Germany (1363 h/year) in 2018 [27]. In terms of working hour regulation, EU member countries are bound to the EU’s Working Time Directive (2003/88/EC). According to this directive, maximum weekly working hours is set to 48 h per week, and the 48-h average is calculated over a reference period of up to 4, 6 or 12 months depending on national legislation and/or collective agreements [28]. Referring to this averaging period of EU countries, some employers in Korea claim that a longer averaging period should be introduced to compensate for the loss of productivity due to the shortened maximum weekly working hours. However, an average of 52 h over 6 months (to be implemented soon in Korea) could make it possible to have workers work extremely long for certain periods of less than 6 months.

In the current study, the gender-stratified analysis showed that the negative mental health impact of variable daily or weekly working hours and/or long working hours was more prominent in men than in women. A previous study in the UK reported that flexible working (flexitime and telework) was associated with staying in employment after the birth of the first child, though it was not statistically significant [29]. In the case of workers having family, considering normative role expectations of women as caregivers and men as breadwinners, women remaining in the labor market might have adjusted or chosen their work patterns to meet the needs of the family. Indeed, among workers with variabily in daily or weekly working hours in our data, women showed a higher prevalence of ‘free choice’ in working hours (37.2%) compared to men (31.7%) (data not shown). Besides mental health, other health outcomes have been linked more strongly with working hours in men than in women in previous Korean studies [30–32]. Korea has a high gender employment gap and that is especially large among workers with tertiary education, showing the highest rank in Organization for Economic Cooperation and Development (OECD) countries [33]. Women might be more likely to leave the labor market than men when their health and well-being is influenced by poor working conditions. Although there was a difference in the strength of association, variable daily or weekly working hours combined with long working hours (> 52 h/w) showed a statistically significant impact on mental health in women as well as in men.

To our knowledge, this is the first study examining the impact of variability in daily or weekly working hours by total weekly working hours on mental health in Korea. Furthermore, restricted study sample to full-time and non-shift workers enabled us to investigate the impact of variable working hours not confounded by precarious work or shift work.

Our study also has several limitations. First, the outcomes were measured by self-reported single item questions. The single self-reported item may underestimate the prevalence of depressive symptoms [34]. Nonetheless, several studies have found that even a single item about depressive symptoms has a strong correlation with clinically diagnostic measures of depression in various populations [35–38]. Also, we controlled for factors such as age and education which might potentially impact the sensitivity of the outcome measure. Working hours were measured by self-report as well; therefore future studies could perform more detailed analysis using objective and elaborate measures of working hours and mental health. Second, due to the cross-sectional design, a causal relationship could not be determined between variable daily or weekly working hours (with/without long working hours) and self-reported mental health problems. Reverse causation, i.e., the possibility that non-depressed workers are better able to arrange for themselves fixed work schedules, could not be ruled out.

## Conclusions

Our study revealed that workers who do not work same number of hours per day or week have a higher risk of depressive symptoms and anxiety than workers with same number of working hours. Although the impact is greater when variable daily or weekly working hours are combined with long working hours, even workers working within permissible maximum hours are adversely impacted by variable working hours. These considerations need to be taken into account in future revisions of the Labor Standards Act.

## Supplementary Information

**Additional file 1: Supplementary Table S1.** Distribution of variable working hours.

## Data Availability

The data that support the findings of this study are available on request from the Occupational Safety and Health Research Institute, Korea Occupational Safety and Health Agency.

## Abbreviations

KWCS: Korean Working Conditions Survey
KOSHA: Korea Occupational Safety and Health Agency
OR: Odds ratio
CI: Confidence interval
EU: European Union
OECD: Organization for Economic Cooperation and Development
