# Supplementary material for: Variability in daily or weekly working hours and self-reported mental health problems in Korea, Korean working condition survey, 2017
Source: Arch Public Health. 2021 Feb 27;79:25. doi: 10.1186/s13690-021-00545-z (PMC7912832; doi:10.1186/s13690-021-00545-z)
Supplement: Supplementary file 1 — Additional file 1: Supplementary Table S1. Distribution of variable working hours. [file 13690_2021_545_MOESM1_ESM.docx]

Supplementary Table S1. Distribution of variable working hours

|  |  | Regular working hours | | Variable daily or weekly working hours | |
| --- | --- | --- | --- | --- | --- |
|  |  | Weighted frequency | % | Weighted frequency | % |
| Total |  | 23279 | 82.1 | 5066 | 17.9 |
| Sex | Men | 13631 | 80.2 | 3372 | 19.8 |
|  | Women | 9648 | 85.1 | 1694 | 14.9 |
| Age | <30 | 3464 | 84.2 | 649 | 15.8 |
|  | 30-39 | 6249 | 82.2 | 1352 | 17.8 |
|  | 40-49 | 6599 | 81.9 | 1456 | 18.1 |
|  | 50-59 | 4921 | 81.1 | 1147 | 18.9 |
|  | 60- | 2046 | 81.6 | 462 | 18.4 |
| Education | <High school | 1178 | 76.9 | 355 | 23.1 |
|  | High school | 6279 | 79.7 | 1602 | 20.3 |
|  | College | 15822 | 83.6 | 3109 | 16.4 |
| Occupation | Manager | 931 | 86.6 | 145 | 13.4 |
|  | Professional | 2582 | 84.1 | 488 | 15.9 |
|  | Technicians and associate professionals | 1541 | 81.6 | 349 | 18.4 |
|  | Clerk | 8250 | 86.4 | 1301 | 13.6 |
|  | Service | 2452 | 82.1 | 534 | 17.9 |
|  | Sales worker | 1973 | 75.5 | 640 | 24.5 |
|  | Agricultural, forestry or fishery | 35 | 59.4 | 24 | 40.6 |
|  | Craft and related trades | 2231 | 77.2 | 659 | 22.8 |
|  | Plant, machine operator or assembler | 1559 | 78.7 | 422 | 21.3 |
|  | Elementary occupation | 1725 | 77.4 | 505 | 22.6 |
| Working hour per week | 31-40 | 13821 | 86.7 | 2113 | 13.3 |
|  | 41-52 | 6614 | 76.3 | 2054 | 23.7 |
|  | 52- | 2844 | 76.0 | 899 | 24.0 |
| Monthly salary | <200 | 5838 | 84.4 | 1076 | 15.6 |
| (10000 KRW) | 200-299 | 7260 | 82.1 | 1587 | 17.9 |
|  | 300-399 | 5621 | 81.6 | 1271 | 18.4 |
|  | 400- | 4560 | 80.1 | 1132 | 19.9 |
| Choice of working hour | Rigid | 20290 | 85.8 | 3369 | 14.2 |
|  | Free choice | 2989 | 63.8 | 1698 | 36.2 |
| Overtime pay | No or N/A | 12650 | 83.4 | 2520 | 16.6 |
|  | Yes | 10629 | 80.7 | 2546 | 19.3 |
